# **Living Related Donor Liver Transplantation with Atrio-Caval Anastomosis of Inferior Vena Cava Graft Stored in Deep-Freeze for Budd-Chiari Syndrome**

**Published:** 2015-02-01

**Authors:** F. Yaylak, V. Ince, B. Barut, B. Unal, M. Kilic, S. Yilmaz

**Affiliations:** Inonu University, Liver Transplantation Institute, Malatya, Turkey

**Keywords:** Budd-Chiari syndrome, Living donors, liver transplantation, Vena cava, inferior, Reconstructive surgical procedures

## Abstract

We have previously reported our experience in inferior vena cava resection and reconstruction techniques during liver transplantation for Budd-Chiari syndrome. Herein, we present on a case that demonstrates the importance of experience in complex vascular reconstruction techniques for living donor liver transplantation. A 15-year-old boy was scheduled for living donor liver transplantation for Budd-Chiari syndrome. Venous occlusion was extended up to the right atrial orifice of the supra-hepatic vena cava. Retro- and supra-hepatic segments of the vena cava was resected. Inferior vena cava graft stored in deep-freeze was available. Venous reconstruction was performed with end-to-end atrio-caval anastomosis. Surgical treatment was completed with the implantation of the right liver lobe donated by the patient’s mother. Post-surgical course was uneventful.

## INTRODUCTION

Budd-Chiari syndrome (BCS) is the occlusion of major hepatic veins (HV) and retro-hepatic inferior vena cava (IVC) [1, 2]. In this rare clinical condition, IVC replacement and orthotopic liver transplantation (deceased donor or living donor) have been shown to successfully treat the condition [2, 3]. For IVC replacement, the fibrotic and/or occluded part of the vein is first resected. Replacement is achieved with patches or grafts. Synthetic, cryopreserved or stored in deep freeze grafts may be used [2, 4, 5].

We have previously published our experience with IVC replacement procedure during living donor liver transplantation (LDLT). We have reported one case with aortic graft for IVC replacement during the surgical management of hydatid cyst-related BCS. The suprarenal subdiaphragmatic segment of the IVC was replaced with cryopreserved aortic graft after resection of the fibrotic vein [4]. The next case was a patient with alveolar hydatid cyst in whom IVC replacement was required due to technical difficulties during the recipient hepatectomy and LDLT. A cryopreserved IVC graft was used for the replacement in this case [6]. In our previous experience, preservation of supra-hepatic vena cava was available. All resections and cava replacement procedures were performed by a senior transplant surgeon. 

Herein, we present the anastomosis of IVC graft, stored in deep freeze, to right atrium for caval replacement in a case with BCS, who were treated with LDLT**.**

## CASE PRESENTATION

A 15-year-old boy with a MELD score of 8 was scheduled for LDLT for chronic liver disease and BCS. Pre-operative doppler ultrasonography demonstrated a normal portal vein. A partial obstruction in vena cava was observed with complete obstruction of the hepatic veins due to thrombosis. Furthermore, extensive ascites was observed. CT demonstrated partial thrombus between the IVC and right atrium; hepatic veins could not be observed. A right liver lobe was donated by the patient’s mother. 

**Figure 1:**
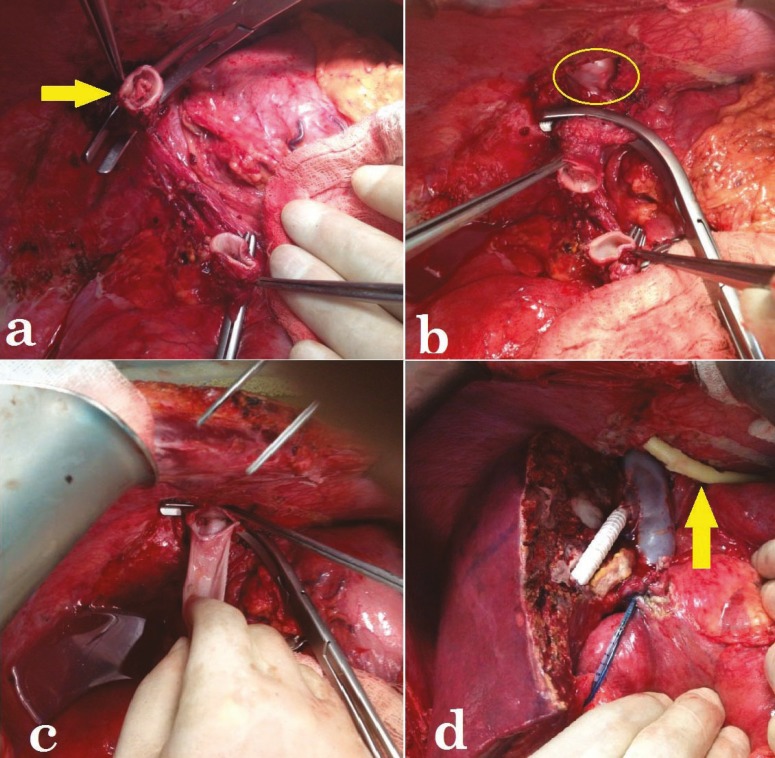
a) The arrow shows the obstruction in the hepatic vein (HV). b) The inferior vena cava (IVC) segment between the clamps was fully mobilized and resected. The ellipse shows the right atrium. c) Reconstruction was performed with stored in deep freeze IVC graft. d) Right hepatic vein of the right liver graft was anastomosed end-to-side to the IVC graft. The arrow shows the Foley catheter placed trans-diaphragmatically into the mediastinum for drainage.

During recipient operation, extensive ascites was observed. Total hepatectomy was performed for the congested and granular liver with preservation of retro-hepatic IVC. The IVC was fibrotic and occluded (Fig 1a). The supra- and infra-segments of IVC were clamped. Distal clamp was placed superior to the renal veins; below and proximal clamp was at the level of right atrium. The IVC segment between the clamps was fully mobilized and resected. Reconstruction was performed with IVC graft stored in deep freeze (Fig 1b-c). The atrio-caval and cava-caval anastomosis were performed. Right hepatic vein of the right liver graft was anastomosed end-to-side to the IVC graft (Fig 1d). Post-operative course was uneventful. Post-operative imaging demonstrated patent IVC graft, hepatic vein and artery.

## DISCUSSION

Orthotopic liver transplantation (deceased donor or living donor) may be the only surgical option for some patients with BCS. However, the extent of venous occlusion determines the extension of the surgical intervention. For patients with profound inferior vena cava occlusion, resection of the vein should be performed and reconstruction is required to supply a patent venous drainage. Cava reconstruction techniques gain importance especially for the living donor recipient patients. Therefore, limitations in the supply of these materials should be considered before scheduling of the LDLT operation for BCS.

Reconstruction of the resected cava requires cryopreserved major vascular grafts or the prosthetic materials [4-7]. However, presence of few reports limits comparison of outcomes with alternative techniques. 

In some cases, veno-venous bypass technique may be required but we could perform hepatic vein anastomosis by total clamping of the IVC in almost all the LDLT operations with significant contribution of the anesthesiologist. Veno-venous bypass was not used in any cases. If the patient could not tolerate total IVC clamping, the hepatic vein anastomosis was used by side clamping of the IVC.

Currently, the need of the patient, presence of an experienced surgeon and the availability of vascular grafts or the prosthetic materials determine the venous patency and the overall results. 

The distal and proximal surgical margins for the resection are primarily determined by the extension of the thrombosis and fibrosis in the occluded vena cava. In our case, thrombosis was extended up to the right atrium. Thus, resection of supra-hepatic cava was needed. The IVC graft stored in deep freeze was available for reconstruction and end-to-end atrio-caval anastomosis was performed by a senior transplant surgeon experienced in advanced vascular reconstruction techniques in liver transplantation. 

This case underlines the importance of the perquisites for surgical treatment of BCS with LDLT. An extended resection of cava up to the right atrium and advanced vascular reconstruction techniques with grafts or prosthesis may be required. Therefore, a transplant surgeon with advanced vascular reconstruction experience is essential to manage such patients. 
